# World Health Organization’s estimates of death related to road traffic crashes and their discrepancy with other countries' national report

**DOI:** 10.5249/jivr.v12i3.1425

**Published:** 2020-08

**Authors:** Alireza Razzaghi, Hamid Soori, Alireza Abadi, Ardeshir Khosravi

**Affiliations:** ^*a*^ Road Traffic Injury Research Center, Tabriz University of Medical Sciences, Tabriz, Iran.; ^*b*^ Safety Promotion and Injury Prevention Research Center, Shahid Beheshti University of Medical Sciences, Tehran, Iran.; ^*c*^ Department of Community Medicine, Faculty of Medicine, Shahid Beheshti University of Medical Sciences, Tehran, Iran.; ^*d*^ Social Department of Health Research Center, Shahid Beheshti University of Medical Sciences, Tehran, Iran.; ^*e*^ Department of Statistics and Informatics, Iranian Ministry of Health and Medical Education, Tehran, Iran.

**Keywords:** Road Traffic Injury, Estimation, Fatal Road Traffic Injury

## Abstract

**Background:**

Due to a lack of effective registry system for road traffic deaths, some international organizations like the World Health Organization provide the estimated number of road traffic deaths. It was shown that there are differences in the number of road traffic deaths between the WHO estimates and national reports even in High-Income Countries. This study aimed to an investigation of reasons for differences between the national reports and world health organization estimates about road traffic deaths.

**Methods:**

This study focus to investigate the World Health Organization reports of Global Status Report for Road Safety in years of 2009, 2013, 2015 and 2018 and related articles about the estimates of deaths related to road traffic crashes and the observed differences between the WHO estimates and national reports.

**Results:**

The findings showed that the observed differences between the WHO estimates and national reports could be due to errors in the road traffic death registration system, errors in the regression model which was used for estimation, proposed variables for estimations, or all of them.

**Conclusions:**

The estimations of WHO about road traffic deaths can be useful for countries especially for those which don’t have the road traffic registry system or the registry system does not meet the quality criteria. These estimates may not be sufficiently robust if disregard for spa-tial differences, the epidemiological pattern of risk factors among the countries, and the type of regression model which was used for estimation.

## Introduction

Road traffic injuries are one of the major public health problems in many countries around the world, especially in low- and middle-income countries (LMICs).^1-3^ One of the weaknesses of road safety management and planning for controlling and decreasing road traffic inju-ries (RTIs) is related to the unavailability of accurate data due to lack of road traffic registry systems. The lack of road traffic mortality registry systems leads to the inability to determine the size and nature of the traffic safety problem. This condition makes it difficult to access accurate information for setting policy goals as well as developing, monitoring and evaluating action plans.^1^

In many high-income countries (HICs), there are high-quality data registry systems (DRSs), which enable countries to identify risk factors. Such registry systems can be applied for implementing the most appropriate and timely interventions for the prevention of road traffic crashes and their related injuries.^1,2,4^ In countries where road traffic registry systems do not exist or are of poor quality, the important step in identifying the situation and risk factors, and also providing appropriate solutions is impaired. In LMICs, it may not be possible to set up a good quality registry system in the short-term. Therefore, there is a need to use estimation methods to gain information about the number of deaths in order to provide management and to take appropriate interventions. 

Regression models are statistical methods that are used to estimate road traffic crashes and traffic-related deaths. These estimation methods are used not only by countries, but also by international organizations, such as the World Health Organization (WHO), for providing the required information and implementing international-level interventions. ^1^ WHO has provided the estimated number and rate of deaths related to road traffic crashes (per 100000 population) in the global status report of road safety (GSRRS) in 2009,^5^ 2013,^6^ 2015^7^ and 2018.^1^

It has been shown that there are differences in the number of road traffic deaths between the WHO estimates and national reports. These differences are seen among countries, but the level of difference is considerable, especially in LMICs. According to previous studies, there may be under-reporting of road traffic-related deaths (RTDs) in these countries.^8-10^ On the other hand, the difference observed in some HICs with a high-quality injury surveillance system raises the question of whether the WHO estimates are likely to be erroneous, and what are the sources of these possible errors? There are some important issues in the differences observed between WHO estimates of deaths related to road traffic crashes and those of the national reports in some countries.^1,5-7^ To the best of our knowledge, no study has investigated the possible errors in WHO estimates. This study aimed to investigate the reasons for the differences observed between the national reports and WHO estimates of road traffic-related deaths. 

## Methods 

The aim of this study was to investigate the WHO reports of GSRRS in the years 2009, 2013, 2015 and 2018, and also to review related articles about the estimates of deaths related to road traffic crashes and the differences observed between WHO estimates and national reports. Every two to three years, WHO publishes road safety reports, which provide information about road safety for countries around the world. Road traffic deaths were estimated for countries by using the negative binomial regression model. At first, there was a fitted model using the data of 86 countries with death registry systems. The completeness criteria for death registration in these countries was 80% or more. The fitted model was used for estimating road traffic deaths for countries. The covariates that were used for estimating road traffic deaths included gross domestic product (GDP), total vehicle per 1000 person, total road per 1000 hectares, national speed limits on rural and urban roads, health system access, working population, percentage of motorbikes, corruption index, national policies for walking and cycling, and the total population. The differences between WHO estimates and national reports were studied in GSRRS.^1^

To determine the reasons for the differences observed between WHO estimates and national reports, all studies about road traffic death registry systems, regression models for estimation of road traffic-related deaths, and risk factors associated with road traffic-related deaths were searched. The following terms were searched in the databases of PubMed, Web of Science and Scopus: estimation, road traffic deaths, under-reporting, national report, and the combination of these keywords. 

## Results and Discussion

In the 2009, 2013, 2015 and 2018 GSRSS, it was shown that there are differences in the number of road traffic-related deaths between the WHO estimates and national reports. Most of the countries in which the differences were observed belonged to the LMICs (Table 1). ^11-13^ These differences could be due to errors in the road traffic death registration systems, errors in the regression model that was used for estimation, proposed variables for the estimations, or all of them. 

**Table 1:** The estimated and reported number of RTDs among some countries around the world in reports of 2013, 2015 and 2018.

GSRRS	2013		2015		2018	
Countries with higher estima-tion than reported number of deaths	NRNRTD^1^	ENRTD^2^	NRNRTD	ENRTD	NRNRTD	ENRTD
Iran	23249	25224	17 994	24 896	15932	16426
Viet Nam	11859	21651	9 845	22 419	8417	24970
Thailand	13365	26312	13 650	24 237	21745	22491
India	130037	231027	137 572	207 551	150785	2990191
Germany	3648	3830	3 339	3 540	581	599
Turkey	5253	8758	4786	6687	7300	9782
Pakistan	30131	26751	9 917	25 781	4448	27582
Nigeria	5279	53339	6 450	35 641	5053	39802
China	70134	275983	62 945	261 367	58022	256180
Egypt	9608	10729	8 701	10 466	8211	9287
Bulgaria	775	776	601	601	708	730
Azerbaijan	1202	1202	1 256	943	759	845
Mexico	17301	16714	17 139	15 062	16039	16725

1 National Reported Number of Road Traffic Deaths. 2 Estimated Number of Road Traffic Deaths by WHO.

**Issues related to road traffic death registration systems**

There are different types of road traffic death registration systems around the world. In HICs, the injury surveillance system provides high-quality information about road traffic-related injuries and deaths. However, in most LMICs, due to the lack of reliable data registry systems, it is not possible to directly and accurately measure road traffic-related injuries and deaths. Therefore, there are some other road traffic death registration systems, which are used for national and international reports.^14^ For instance, in countries like India and China a reliable registration system is not available. However, both countries have the sample registration system (SRS) that provides an estimation of RTDs.^15^ In many African countries with no vital registration systems, the information resources of cemeteries and the population and health care networks provide the required information about RTDs. Many African cities have cemeteries in which the cause of death is usually recorded for forensic reasons, while the population and health care network information source reports the cause of death in rural areas of Africa.^16^

The death registration systems in LMICs often have local coverage that can only provide reliable data for the covered regions. Therefore, the coverage of death recording data may not be complete in these countries. Moreover, the quality of registration of death causes may be inappropriate and thus lead to incorrect information.^12^ Death registration systems typically use the International Classification of Diseases (ICD)-10 framework for coding, which creates a more detailed set of codes for recording deaths. However, detailed information may not be determined for classification. In the national estimation of RTDs, the types of road users may be undetermined. For instance, data may be recorded as a road user instead of determining the pedestrians, drivers, occupants, and cyclists. ^2^

The findings of related studies show that there is under-reporting of RTDs. Results of a study in Karachi, Pakistan showed that the under-reporting of RTDs in hospital registry systems was about 20%,^8^ while the WHO estimated number of RTDs was about 6 times higher than that of national reports.^1^ The number of RTDs in Iran’s national report was lower than the estimated number of the WHO in the GSRRS reported in 2009, 2013, 2015 and 2018. These estimations can be useful for countries, especially for those that do not have road traffic registry systems or for countries in which the registry system does not meet the quality criteria such as completeness and coverage. Countries in which completeness of death registration data was 80% or more were considered as countries meeting the quality criteria. According to the 2015 GSRRS, the number of RTDs was approximately 28% lower than the estimated number in Iran (17994 versus 24894).^17^ This difference in the 2013 report was about 8% (23249 versus 25224),^6^ and in the 2009 report, it was approximately 11% (22918 versus 25491).^5^ The results of some studies indicate that there is under-reporting of RTDs in Iran too (7, 8, 14, 18). However, the amount of discrepancy reported in these studies differs from that of the WHO estimate. The findings of related studies that have been conducted in local areas show that there is 12-16% under-reporting of the registration of road traffic deaths.^10^ These differences are also seen among other countries, especially LMICs. For example, based on the 2015 GSRRS, this difference is about 66% in Vietnam, 3% in Germany, 22% in Egypt, 26% in Turkey, and 88% in China (Table 1). The remarkable note is that some of these countries are categorized as countries with high-quality registry systems for RTDs by the WHO, which were entered for fitting the regression model. ^1,5,6^

**Issues related to estimation of RTDs based on regression models**

Data on road traffic crashes are classified as count data and so, count regression models are used for modeling and estimating. Several regression models are used for data on road traffic crashes,^18^ some of which are presented in Table 2 with their advantages and disadvantages. 

**Table 2:** Advantages and disadvantages of some models for analyzing crash-frequency data.

Model	Advantages	Disadvantages
**Poisson**	- Basic model for count data	- Cannot account the under and over-dispersion
	- Easy to use	- Influenced by low sample mean and bias of small sample size
**Negative binomial/ Pois-son gamma**	- Easy for estimation	- Cannot account the under-dispersion
	- Can account the over-dispersion	- Influenced by low sample mean and bias of small sample size
**Zero-inflated Poisson and negative binomial**	- Can use for the data with large number of zero-observation crash	- It is threatening by theoretical inconsistency re-lated to low sample mean and bias of small sam-ple size
**Conway-Maxwell-Poisson**	- Can be used for over and under-dispersion or combina-tion of both	- It is negatively influenced low sample mean and bias of small sample size
		- No available the multivariate extension
**Generalized estimating equation models**	- Can handle the temporal correlation	- Sensitive to missing data
**Random-effects models**	- Can handle the temporal and spatial correlation	- The transforming data to other dataset is not easy.

WHO uses the negative binomial model (NBM) for estimating RTDs. In the 2013, 2015, and 2018 reports of GSRRS, countries were classified based on the quality of registry systems as indicated by the completeness criteria. Countries with a high quality road traffic death registration system were defined as countries with a completeness of at least 80% for the study year or an average completeness of 80% or higher for the last 10 years, including the study year.^1^ The fitted model was extracted based on the estimation model of the WHO. In this model, the values of β were applied for the estimation of RTDs of countries that did not meet the completeness criteria.^1^

There are several count regression models; each of them having special prerequisites. At this point, this question comes to mind: is the negative binomial regression model the most appropriate among all the available models for count data? The basic model for count data is the Poisson regression model and the main prerequisite for using this model is the equality of mean and variance for the dependent variable. If this condition is not met, the estimates made might be prone to error. The zero-inflated Poisson distribution models are used if the data contains an excess of zero counts.^19,20^

If the data contains a layer (surface) variable, the multilevel Poisson model should be used.^10^ Considering the features of road traffic data and the existence of over-dispersed data, the NBM is an appropriate model for traffic crash-related studies; thus, the use of this model has extended rapidly among researchers.^21^ It should be noted that the negative binomial regression model is restricted to under-dispersion conditions.^22^ It has been shown that in under-dispersion conditions , the flexibility of the Conway-Maxwell Poisson regression model in traffic accident modeling is better than the NBM. ^23,24^ Estimating RTDs at the international level may be different to estimating those at the national and local levels, which might be likely due to spatial and temporal differences.^25-27^

It seems that in the estimation of RTDs by the WHO, no attention has been paid to spatial dependence between countries of different regions. It was shown that the failure to consider spatial dependence when using the NBM distorts the results of the analysis.^18,28^ One of the reasons for using the NBM is over-dispersion of data; however, this model does not consider spatial dependence (autocorrelation effects) in the analysis. In the WHO estimate, a fitted model was extracted based on the estimating model with data from countries that were mainly high-income European countries. The values of β in the fitted model were then applied for the estimation of RTDs of other countries around the world.^1^ The NBM is a non-spatial model and so, it is not recommended for data with spatial dependence.^8^ However, the existence of spatial dependence is not determined in the WHO estimate and it is not mentioned in the GSRRS reports. 

**Issues related to the pattern of risk factors in different countries**

To prioritize and implement effective interventions, there is a need to have adequate knowledge about the risk factors associated with RTDs, which are be classified into four main categories, including human factors, vehicle-related factors, road-related factors, and environmental factors. The pattern of road traffic injries is different across countries around the world. As with traffic crashes and injuries, the pattern of risk factors for RTDs is also different. In a systematic review, it was shown that similar to the difference in the pattern of road traffic crashes, the pattern of risk factors was also different. For instanse, obesity was reported as a risk factor for road traffic-related deaths in the United States and Europe countries.^29^ In southeastern Asia and the Western Pacific region, old age was reported as a main risk factor for road traffic-related deaths among motorcylists.^29^ In this study, it was concluded that not paying attention to the differences in risk factors associated with RTDs can affect the results of estimations by WHO.^29^

## Conclusion

The estimations of WHO about road traffic deaths can be useful for countries especially for those which don’t have the road traffic registry system or the registry system does not meet the quality criteria. These estimates may not be sufficiently robust if disregard for spatial differences, the epidemiological pattern of risk factors among the countries, and the type of regression model which was used for estimation.

**Aknowledgment**

All the people who helped us in this study are thanked for their cooperation.
